# Bladder-sparing therapy in a case report of huge muscle-invasive bladder IMT treated with 1470 nm diode laser en bloc resection followed by laparoscopic partial cystectomy

**DOI:** 10.3389/fonc.2025.1519676

**Published:** 2025-02-06

**Authors:** Junhao Chu, Huisheng Yuan, Zhihui Zhang, Jiajun Kan, Shishuai Duan, Zilong Wang, Muwen Wang

**Affiliations:** ^1^ Department of Urology, Shandong Provincial Hospital Affiliated to Shandong First Medical University, Jinan, China; ^2^ Department of Andrology, The Seventh Affiliated Hospital, Sun Yat-sen University, Shenzhen, China; ^3^ Department of Urology, Shandong Provincial Hospital, Cheeloo College of Medicine, Shandong University, Jinan, China; ^4^ Scientific Research Center, The Seventh Affiliated Hospital, Sun Yat-sen University, Shenzhen, China

**Keywords:** inflammatory myofibroblastic tumor, muscle invasion, transurethral en bloc resection of bladder tumor, laparoscopic partial cystectomy, bladder tumor, 1470 nm laser, case report

## Abstract

**Background:**

Bladder inflammatory myofibroblastic tumor (IMT) is a rare intermediate malignancy. Muscle-invasive bladder IMT is associated with a high risk of recurrence and metastasis, and bladder-sparing treatments for this condition are still under exploration. This case aims to evaluate the therapeutic efficacy of 1470 nm diode laser transurethral en bloc resection (ERBT) followed by laparoscopic partial cystectomy in the treatment of muscle-invasive bladder IMT.

**Methods and results:**

A 23-year-old male patient presented with painless terminal gross hematuria and was treated at Shandong Provincial Hospital of Shandong First Medical University. Computed tomography urography (CTU) and magnetic resonance imaging (MRI) identified a large tumor on the anterior bladder wall with muscle layer invasion, measuring approximately 5.0 × 3.9 × 4.3 cm. The patient underwent 1470 nm laser ERBT, followed by laparoscopic partial cystectomy 35 days later. Pathological examination following 1470 nm laser resection confirmed the diagnosis of an IMT with malignant potential, showing anaplastic lymphoma kinase (ALK) positivity, a Ki-67 index of 20% in hotspot regions, and ALK gene rearrangement detected by fluorescence *in situ* hybridization (FISH). Pathology after the secondary laparoscopic partial cystectomy showed tumor invasion into the superficial muscle layer, with negative margins at the resection site. MRI and cystoscopy showed no recurrence during 1 year follow-up.

**Conclusion:**

This case presents a patient with a huge muscle-invasive bladder IMT who received bladder-sparing therapy through 1470 nm diode laser ERBT followed by laparoscopic partial cystectomy. During subsequent follow-ups, the patient showed good recovery with no signs of recurrence, providing a promising treatment concept for bladder-sparing therapy in muscle-invasive bladder IMT.

## 1 Introduction

Bladder inflammatory myofibroblastic tumor (IMT) is a rare intermediate-grade malignancy arising from mesenchymal tissues (1, 2). Since Roth’s initial report of bladder IMT in 1980 (3), only less than 100 cases have been reported worldwide (2). Due to its rarity, the risk factors for bladder IMT remain not fully elucidated. Nonetheless, factors such as smoking, minor trauma, and conditions associated with IgG4 have been proposed as potential contributors to its pathogenesis (4, 5). The most frequent initial manifestation of bladder IMT is painless gross hematuria, which is often accompanied by urinary symptoms including frequency, urgency, and dysuria (6).

The primary treatment for bladder IMT is surgical resection (4). Given the tumor’s primary location, size, ages of the patients, and the risks associated with various surgical procedures, selecting a safe and appropriate surgical approach is crucial in the management of bladder IMT. In our previous study (7), we reported a 42-year-old male patient with a huge non-muscle-invasive bladder IMT that was successfully treated with en bloc resection of the bladder tumor (ERBT) using a 1470 nm diode laser, followed by a secondary transurethral resection. This provides a safe and effective surgery option for non-muscle-invasive bladder IMT.

Reports on the treatment of muscle-invasive bladder IMT remain scarce. Typically, radical cystectomy (RC) is traditionally considered the gold standard for muscle-invasive bladder tumors (8). However, due to the high complication rates and the profound effect on patients’ quality of life, bladder-sparing approaches have gained prominence in recent years (9).

In this report, we present a 23-year-old male patient with a huge muscle-invasive bladder IMT who was treated with ERBT using a 1470 nm diode laser, followed by laparoscopic partial cystectomy within six weeks. Over a one-year follow-up period, MRI and cystoscopic examinations showed no recurrence. The objective of this case report was to provide a promising treatment concept for bladder-sparing therapy in muscle-invasive bladder IMT.

## 2 Case presentation

### 2.1 Preoperative condition

**Figure 1:**
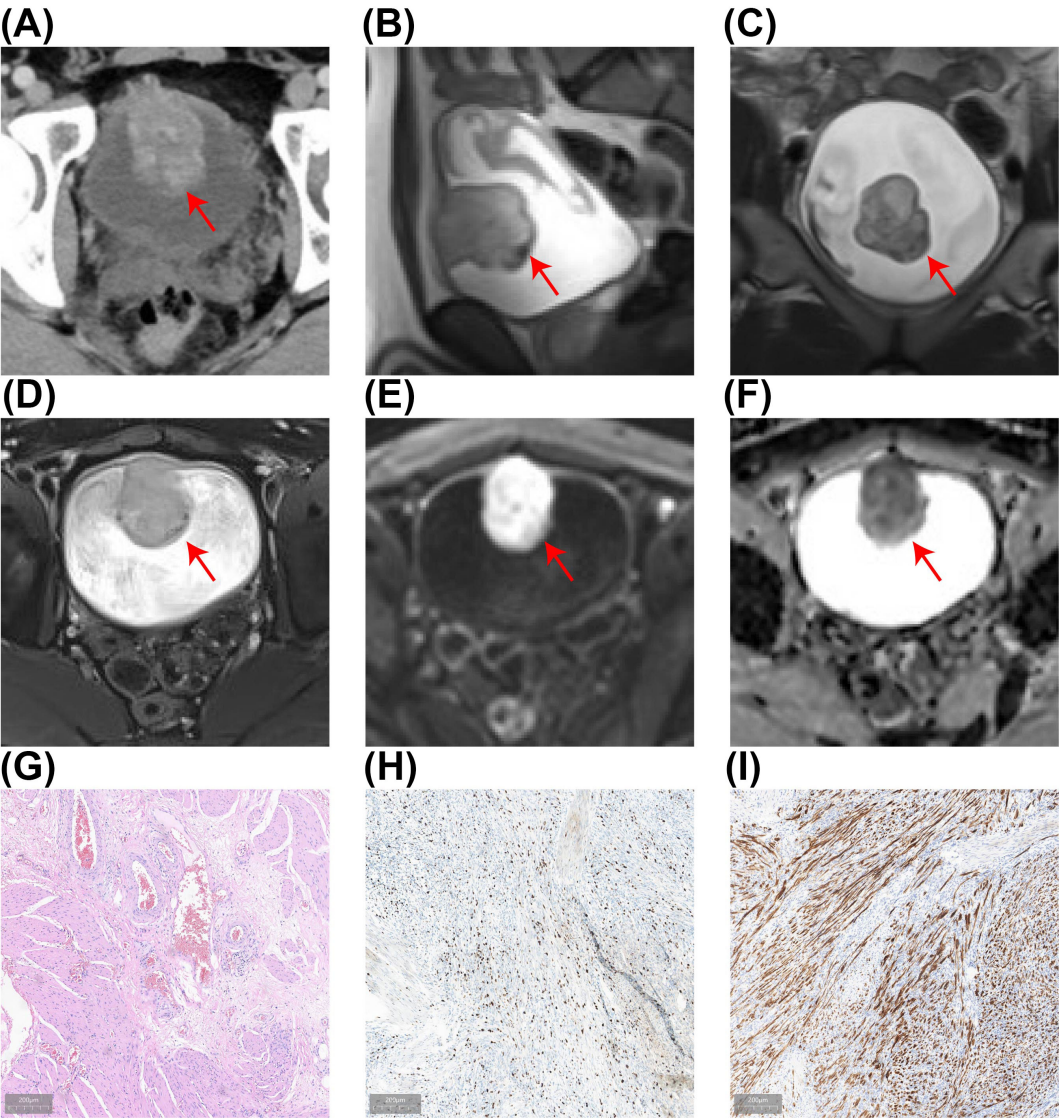
The results of preoperative examination. **(A)** CTU revealing a mass lesion (5.0 × 4.3 cm) in the anterior bladder wall with heterogeneous enhancement during the excretory phase, suggesting potential muscle layer infiltration. **(B-F)** MRI revealed a bladder tumor (5.0 × 3.9 × 4.3 cm) with mildly elevated T2-weighted signals in sagittal **(B)**, coronal **(C)**, and transverse **(D)** views, with significant diffusion restriction visible on DWI **(E)** and ADC **(F)**. **(G)** HE of the bladder biopsy showing IMT with HE staining. **(H)** The Ki-67 positivity index is approximately 20%. **(I)** ALK-positive results on IHC.

A 23-year-old male patient was admitted to the Department of Urology on January 18, 2024, due to experiencing painless terminal gross hematuria for a week. The patient had a 5-year history of smoking, averaging 10 cigarettes per day, with no history of chronic diseases such as hypertension or diabetes and no family history of hereditary diseases. Clinical laboratory tests, including routine blood tests, serum electrolytes, liver function, lipid profile, and renal function were all within normal limits. Urinalysis revealed red blood cells at 1511.8/HPF (normal range ≤ 3/HPF). Computed tomography urography (CTU), magnetic resonance imaging (MRI) and cystoscopic examination identified a large tumor on the anterior bladder wall with muscle layer invasion, measuring approximately 5.0 × 3.9 × 4.3 cm (**Figures 1A–D**; **Supplementary Video S1**). Diffusion-Weighted Imaging (DWI) and Apparent Diffusion Coefficient (ADC) demonstrated significant diffusion restriction (**Figures 1E, F**). Preoperative bladder biopsy pathology indicated an inflammatory myofibroblastic tumor (IMT), with immunohistochemistry (IHC) showing Ki-67 positivity (hotspot area 20%) and positivity for anaplastic lymphoma kinase (ALK) (**Figures 1G–I**). Based on the biopsy pathology and imaging findings, the preoperative diagnosis was a huge muscle-invasive IMT of the bladder. Given the high Ki-67 expression and myometrial invasion, we opted for a two-stage surgical approach to prevent tumor cell dissemination and diminish the extent of surgical resection associated with direct laparoscopic partial cystectomy, thereby improving bladder preservation rates. Initially, we performed an en bloc resection of the bladder tumor (ERBT) to achieve maximum tumor removal, followed by laparoscopic partial cystectomy six weeks later.

### 2.2 En bloc resection of bladder IMT and postoperative pathological results

**Figure 2:**
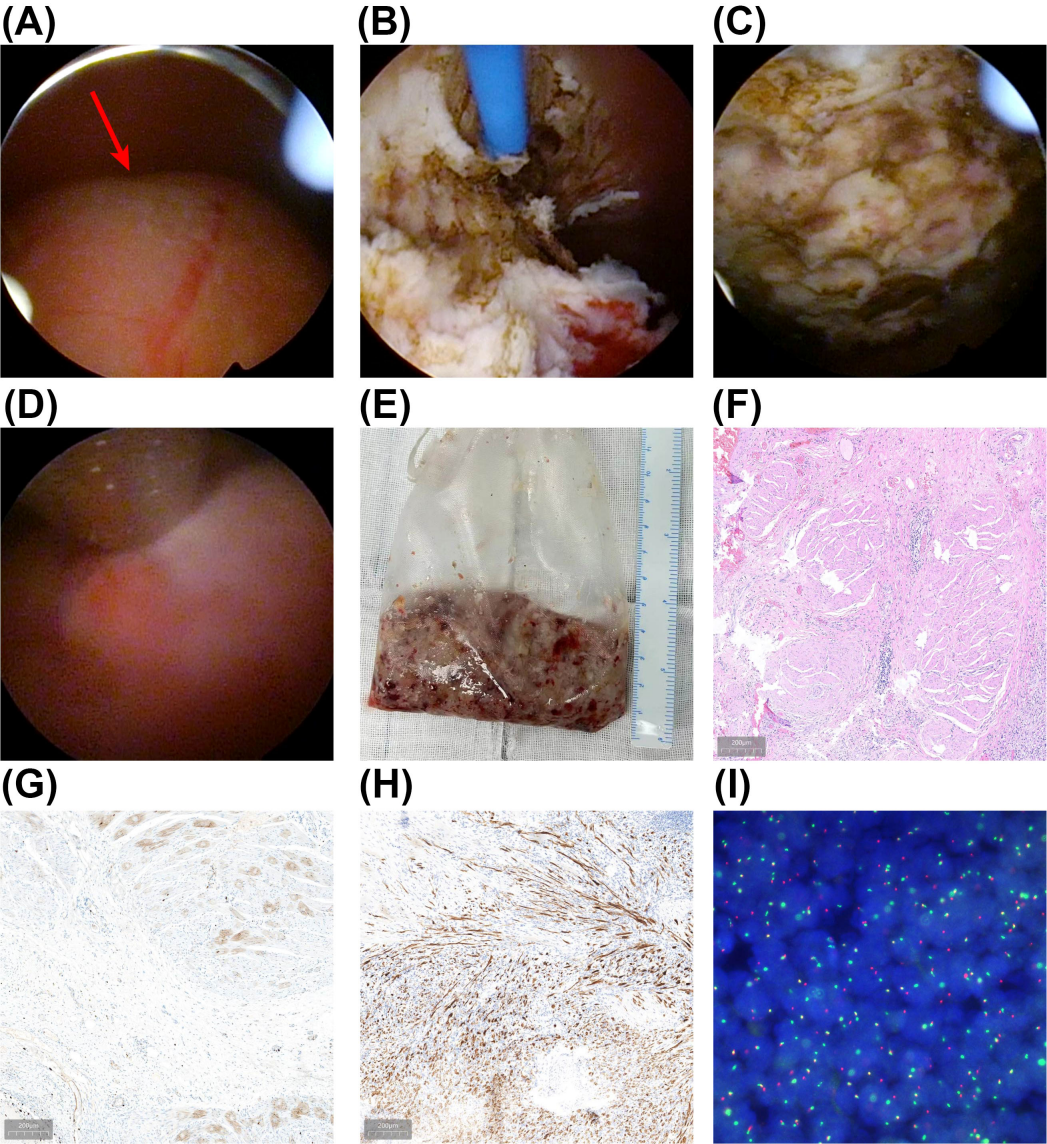
En Bloc Resection of Bladder Tumor and Pathological Findings. **(A)** Intraoperative view displaying a large polypoid inflammatory myofibroblastic tumor (IMT) of the bladder. **(B)** The tumor is being resected with a 1470 nm semiconductor laser, following the circular muscle layer marking toward the tumor base. **(C)** The surgical site post-complete tumor resection. **(D, E)** The excised tumor was removed using a tissue morcellator. **(F)** Initial postoperative pathological results indicating bladder IMT, visualized with hematoxylin-eosin (HE) staining. **(G, H)** Immunohistochemical (IHC) analysis shows positive results for a Ki-67 proliferation index of 15% **(G)** and anaplastic lymphoma kinase (ALK) **(H)**. **(I)** Fluorescence *in situ* hybridization (FISH) confirms the presence of positive ALK gene rearrangement.

After thorough preoperative preparation, the patient underwent en bloc resection of the bladder tumor (ERBT) on January 23, 2024. The procedure utilized a 1470 nm diode laser treatment system (Wuhan Qizhi Laser Technology Co., Ltd.), with a cutting power set at 100 W and coagulation power at 30 W, using sterile saline as the irrigation fluid. Energy was delivered via a 600 µm fiber through a 26 F continuous-flow cystoscope (Batch number DQH-111, Hawk, Hangzhou, China). General anesthesia was administered, and the patient was positioned in the lithotomy position. The lead surgeon lubricated the obturator with sterile paraffin oil, inserted it through the urethra, and removed the inner core before introducing the cystoscope. A thorough cystoscopic examination revealed a polypoid tumor approximately 5 × 4 cm on the anterior bladder wall (**Figure 2A**). After confirming the tumor’s location, a 1470 nm diode laser fiber was introduced. The tumor’s boundary was marked in a circular manner about 2 cm from the edge, cutting down to the detrusor layer. This allowed the tumor to retract towards the center after releasing the mucosa and submucosa while also pre-coagulating the surrounding exposed submucosal vessels.

Subsequently, along the marked muscle layer, either an antegrade or retrograde approach was used to excise the tumor, employing a combination of sharp and blunt dissection towards the base. The laser was used to dissect the tumor’s base, visually separating the myofibers until the entire tumor was removed, thus obtaining a complete pathological specimen, which was extracted using a tissue morcellator (**Figures 2B–E**; **Supplementary Video S2**). The incision surface was carefully examined, and laser coagulation was used for hemostasis.

Intraoperative blood loss was minimal, and the surgery proceeded smoothly without complications such as obturator nerve reflex, bladder perforation, or significant bleeding. An F20 three-way catheter was retained postoperatively, and continuous bladder irrigation was performed for two days. The postoperative pathological results indicated bladder IMT (**Figure 2**), with a small amount of tumor present in the bladder base tissue. Immunohistochemistry (IHC) results showed Ki-67 positivity (15%), with positive staining for smooth muscle actin (SMA) and anaplastic lymphoma kinase (ALK) in the bladder IMT. Fluorescence *in situ* hybridization (FISH) confirmed positive ALK gene rearrangement (**Figures 2F–I**).

### 2.3 Laparoscopic partial cystectomy and postoperative pathology

**Figure 3:**
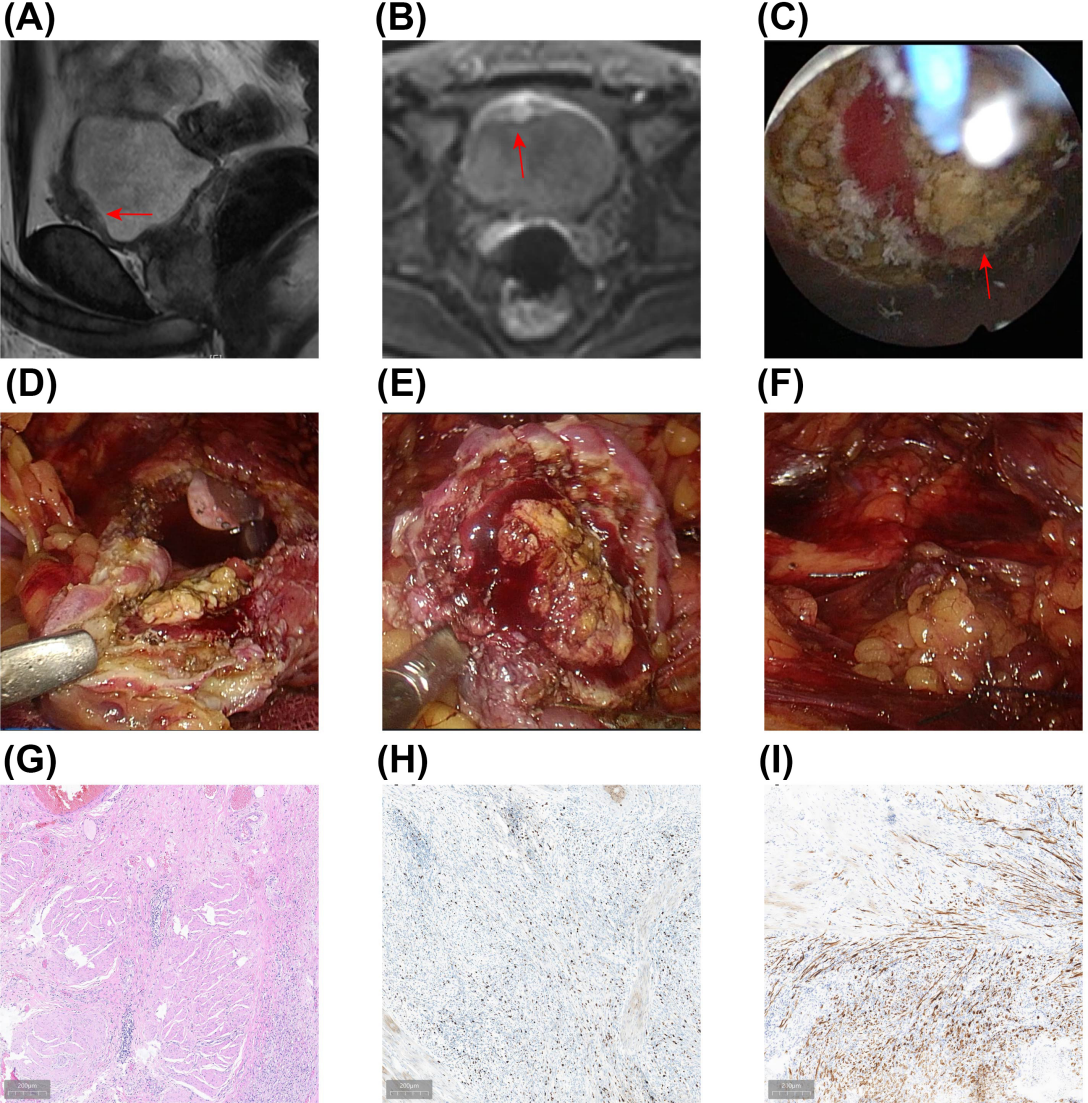
Results of the Secondary Laparoscopic Partial Cystectomy and Postoperative Pathology. **(A, B)** Preoperative MRI demonstrated irregular thickening of the anterior bladder wall **(A)**, with no significant diffusion restriction observed on DWI **(B)**. **(C)** A 1470 nm semiconductor laser was utilized to mark the resection margin for the laparoscopic partial cystectomy. **(D-F)** Under laparoscopy, the original surgical scar was completely excised 1.5 cm beyond the marked margin, followed by suturing of the bladder. **(G)** Postoperative HE staining reveals bladder IMT affecting the superficial muscle layer of the bladder. **(H, I)** IHC results indicate Ki-67 positive (15%) **(H)** and ALK-positive **(I)**.

The patient underwent laparoscopic partial cystectomy on February 27, 2024. Preoperative MRI revealed localized thickening of the anterior bladder wall, with the thickest part measuring approximately 1.4 cm and showing a slightly elongated T2 signal, without significant diffusion restriction on DWI (**Figures 3A, B**). Under general anesthesia, the patient was positioned in the lithotomy position, and a cystoscope was inserted. A comprehensive cystoscopic examination revealed surgical scars and an area of inflammatory edema on the anterior bladder wall. An approximately 1 cm circular margin was marked around the scar and edematous area as the resection boundary for the laparoscopic partial cystectomy (**Figure 3C**). Tissue was vaporized along the marking down to the muscular layer (**Figure 3D**), and an F20 triple-lumen catheter was left in place after cystoscope withdrawal.

The patient was then repositioned in a head-down, foot-up posture with the pelvis elevated, and the surgical field was disinfected and draped. Trocar insertion points were selected at the upper umbilical region, the intersection of the middle and inner thirds of the line connecting the umbilicus and the left/right anterior superior iliac spines, and the middle and outer thirds of the line connecting the umbilicus and the right anterior superior iliac spine. Pneumoperitoneum was established using a Veress needle set to 15 mmHg pressure. A 10 mm trocar was inserted at the umbilicus for an observation scope, while other trocars were inserted into the remaining puncture sites. After inserting the observation scope, a thorough examination of the abdominal organs was performed, and the bladder boundaries were identified. The peritoneum at the base of the abdomen was opened with an ultrasonic scalpel, carefully freeing the anterior dome of the bladder. After bladder distention, a full-thickness excision of the original surgical scar, 1.5 cm external to the laser-marked margin, was performed (**Figure 3E**). The excised tissue was placed in a specimen bag. The bladder was then sutured in two layers using 2-0 V-Lok sutures (**Figure 3F**). A latex drain was placed behind the pubic bone and exited through a trocar port. After bladder filling, no leakage was observed (**Supplementary Video S3**).

After specimen removal, the surgical area was inspected for bleeding points. Once the correct placement of instruments and gauze was confirmed, the incision was closed in layers. Postoperative pathology reported bladder IMT involving the superficial muscle layer of the bladder, with no tumor found at the bladder’s margins or base. Immunohistochemistry results showed ALK positivity and Ki-67 positivity (15%) (**Figures 3G–I**).

### 2.4 Postoperative follow-up conditions

**Figure 4:**
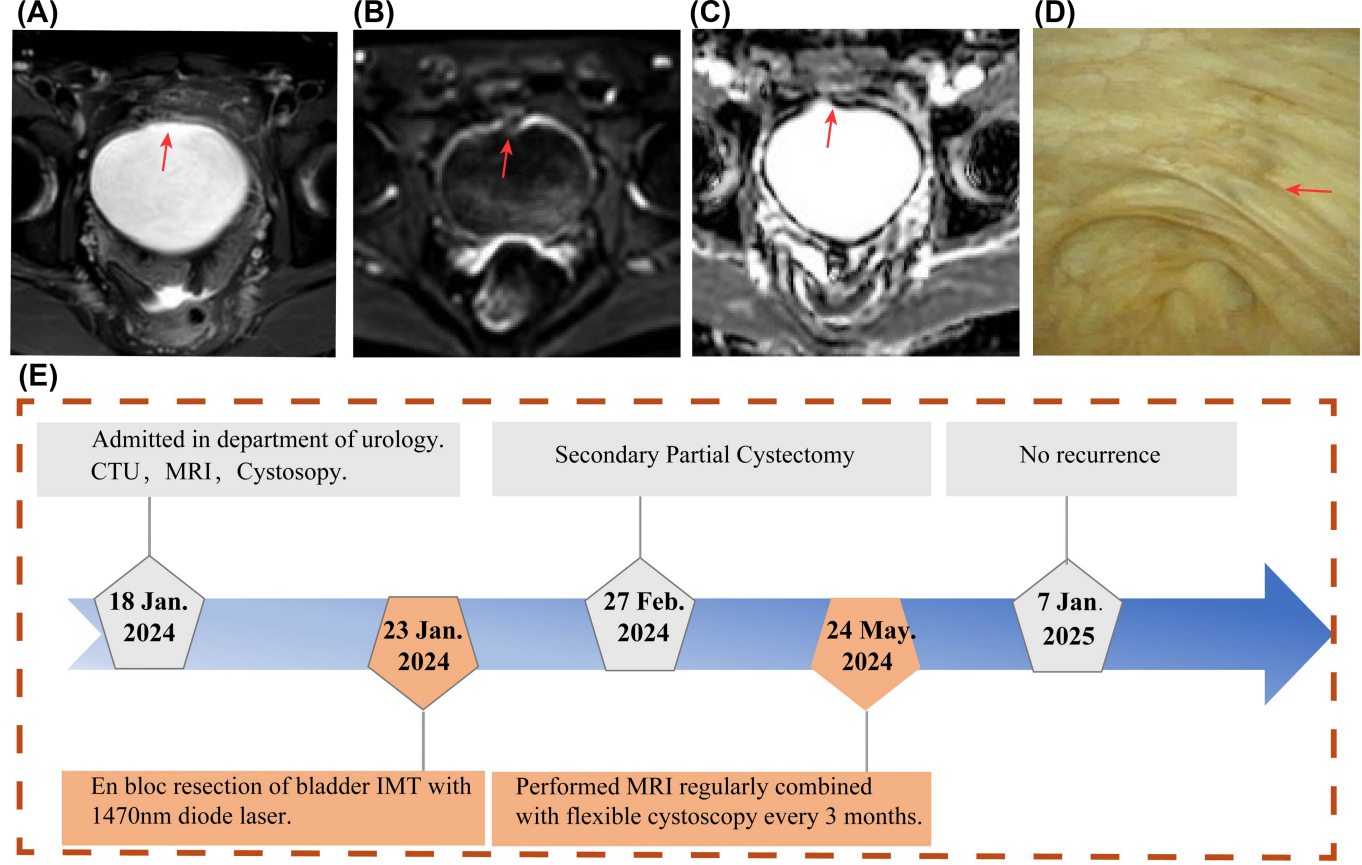
Postoperative Follow-up Examination Results. **(A-C)** One year after surgery, MRI scans show no evidence of tumor recurrence in the axial views **(A)**, with no diffusion restriction observed on DWI **(B)** and ADC **(C)**. **(D)** Flexible cystoscopy performed postoperatively demonstrated the healing status of the surgical wound. **(E)** A timeline flowchart outlining the diagnosis and treatment process.

The bladder MRI and flexible cystoscopy revealed no tumor recurrence or other abnormalities during 1-year follow-up (**Figures 4A–D**). The patient has undergone cystoscopic follow-up every 3 months and remains in good condition with no evidence of tumor recurrence (**Supplementary Video S4**). Throughout a one-year follow-up period, the patient expressed satisfaction with the treatment outcomes. The timeline for the diagnosis and treatment plan is shown in the flowchart in **Figure 4E**.

## 3 Discussion

This study presents a case of a huge (>5 cm) muscle-invasive bladder inflammatory myofibroblastic tumor (IMT) successfully treated with en bloc resection of the bladder tumor (ERBT) using a 1470 nm diode laser, followed by a secondary laparoscopic partial cystectomy. Immunohistochemistry (IHC) revealed a Ki-67 index of 15%, with positive staining for anaplastic lymphoma kinase (ALK) and fluorescence *in situ* hybridization (FISH) confirmation of ALK gene rearrangement. To prevent recurrence and ensure complete tumor removal, a secondary laparoscopic partial cystectomy was performed within six weeks. The 1470 nm diode laser was innovatively used to mark the resection margin, followed by full-thickness excision of the previous surgical scar. Postoperative pathology confirmed tumor involvement in the superficial muscle layer but no residual tumor at the resection margins. The patient remained recurrence-free after 1 year of regular MRI and cystoscopic follow-up.

IMT is a rare tumor composed primarily of spindle-shaped fibroblasts and myofibroblasts, accompanied by inflammatory cells such as lymphocytes, plasma cells, and eosinophils (10). Due to its intermediate malignancy, invasiveness, and recurrence potential, the World Health Organization (WHO) classifies IMT as a tumor of intermediate grade (1, 5). IMT typically affects children and young adults, most commonly in the lungs, head, and neck, but occurrences in the bladder are exceptionally rare (11–14).

Surgical resection is the preferred treatment for bladder IMT, with the standard approach being complete excision with negative margins (2, 4, 15). Radical cystectomy (RC) is the gold standard for muscle-invasive bladder tumors (8). The 2003 edition of the Chinese Guidelines for Diagnosis and Treatment of Urological and Andrological Diseases states that radical cystectomy is a high-risk surgery, with perioperative complications ranging from 28% to 64% and a mortality rate of 2.5% to 2.7%. Additionally, urinary diversion following radical cystectomy severely affects patients’ quality of life. As a result, bladder-sparing therapies have gained increasing attention in recent years (9, 16). In this case, the 23-year-old patient was not a candidate for RC, and traditional transurethral resection of the bladder tumor (TURBT) was unlikely to achieve complete excision with clear margins. Laparoscopic partial cystectomy was chosen instead, but it carries the risk of tumor implantation and metastasis within the abdominal cavity (17). After careful consideration, we opted for a two-step approach involving ERBT using a 1470 nm diode laser, followed by secondary laparoscopic partial cystectomy to ensure a tumor-free surgical environment.

TURBT uses monopolar or bipolar current and segmental resection techniques, which can lead to complications such as bleeding, obturator nerve reflex, and bladder perforation. Advances in laser technology have led to the use of various lasers for tumor resection, and compared to traditional TURBT, the 1470 nm diode laser for en bloc resection offers significant benefits. These include reduced intraoperative bleeding, shorter postoperative bladder irrigation times, fewer complications, and faster recovery (18). In this study, the patient was marked by a 1470 nm diode laser combined with a tissue morcellation system for complete tumor excision. Six weeks later, laparoscopic partial cystectomy was performed, with the 1470 nm diode laser used to precisely mark the incision line, ensuring complete excision with negative margins and minimizing the risk of tumor dissemination and metastasis. Given these advantages, the combination of 1470 nm diode laser ERBT and a secondary laparoscopic partial cystectomy may be considered an effective treatment option for large muscle-invasive bladder IMT.

Bladder IMT is a rare tumor with nonspecific clinical manifestations, often mistaken for bladder cancer (5). Unlike bladder cancer, IMT typically appears as a smooth, broad-based lesion on imaging, rather than a papillary mass (19). Cystoscopy typically reveals a solitary, polypoid intraluminal mass, which helps differentiate it from bladder cancer (20). However, the gold standard for diagnosis remains pathological examination and immunohistochemistry (21). Therefore, preoperative cystoscopy and biopsy are crucial for the accurate diagnosis of bladder IMT.

ALK is a transmembrane tyrosine kinase encoded by the ALK gene on chromosome 2, playing a critical role in the pathogenesis of IMT (21). About 50% of IMTs display ALK gene rearrangements, primarily in the form of translocations, resulting in abnormal ALK fusion protein expression (22). The most common method for detecting these rearrangements is fluorescence *in situ* hybridization (FISH), with ALK positivity identified by the presence of ALK immunopositivity or FISH-detected ALK rearrangement in at least 15% of tumor cells (21). For patients who test negative for both ALK and FISH, next-generation sequencing (NGS) can provide a definitive diagnosis, offering insights into kinase fusions and identifying specific fusion partners (23). NGS has proven to be more reliable than IHC for diagnosing ALK fusion-positive IMT (24).

Ki-67 serves as a marker of tumor cell proliferation and malignancy, with a high Ki-67 index often linked to increased risks of recurrence, progression, and decreased survival rates (25). In this case, the patient had a Ki-67 index of 20% and exhibited muscle layer infiltration, indicating a heightened risk of recurrence. Consequently, a combination of 1470 nm diode ERBT and laparoscopic partial cystectomy was performed to ensure complete tumor removal while adhering to the principle of achieving tumor-free margins.

This study has the following limitation: Although 1470 nm laser en bloc resection of bladder tumors (ERBT) combined with laparoscopic partial cystectomy is feasible, the follow-up period is insufficient to assess the prognosis of muscle-invasive bladder IMT.

## 4 Conclusion

Bladder IMT is a rare tumor that can exhibit malignant potential. The primary treatment strategy involves surgical resection with negative margins to preserve bladder function. For huge, muscle-invasive cases of bladder IMT, the combination of 1470 nm diode laser ERBT and laparoscopic partial cystectomy effectively prevents tumor dissemination and metastasis, preserves bladder function, and significantly improves the patient’s quality of life.

## Data Availability

The raw data supporting the conclusions of this article will be made available by the authors, without undue reservation.

## Supplementary material

Supplementary Video 1 Preoperative flexible cystoscopy examination.

Supplementary Video 2 En bloc resection of bladder Inflammatory Myofibroblastic Tumor using a 1470 nm diode laser.

Supplementary Video 3 Laparoscopic partial cystectomy for bladder inflammatory myofibroblastic tumor.

Supplementary Video 4 Postoperative flexible cystoscopy examination.

## References

[1] Sbaraglia M Bellan E Dei Tos AP . The 2020 WHO Classification of Soft Tissue Tumours: news and perspectives. Pathologica. (2021) 113:70–84. doi: 10.32074/1591-951X-213 33179614 PMC8167394

[2] Rich BS Fishbein J Lautz T Rubalcava NS Kartal T Newman E . Inflammatory myofibroblastic tumor: A multi-institutional study from the Pediatric Surgical Oncology Research Collaborative. Int J Cancer. (2022) 151:1059–67. doi: 10.1002/ijc.v151.7 35604778

[3] Roth JA . Reactive pseudosarcomatous response in urinary bladder. Urology. (1980) 16:635–7. doi: 10.1016/0090-4295(80)90578-6 7445316

[4] Gros L Dei Tos AP Jones RL Digklia A . Inflammatory myofibroblastic tumour: state of the art. Cancers (Basel). (2022) 14:3. doi: 10.3390/cancers14153662 PMC9367282 35954326

[5] Wang QA Chen HW Wu RC Wu CE . Update of diagnosis and targeted therapy for ALK(+) inflammation myofibroblastic tumor. Curr Treat Options Oncol. (2023) 24:1683–702. doi: 10.1007/s11864-023-01144-6 PMC10781869 37938503

[6] Chen C Huang M He H Wu S Liu M He J . Inflammatory myofibroblastic tumor of the urinary bladder: an 11-year retrospective study from a single center. Front Med (Lausanne). (2022) 9:831952. doi: 10.3389/fmed.2022.831952 35308527 PMC8928161

[7] Yuan H Wang Z Sun J Chu J Duan S Wang M . A rare huge bladder inflammatory myofibroblastic tumor treated by en bloc resection with diode laser: a case report and literature review. Front Oncol. (2024) 14. doi: 10.3389/fonc.2024.1327899 PMC10961466 38529377

[8] Lenis AT Lec PM Chamie K Mshs MD . Bladder cancer. Jama. (2020) 324:5. doi: 10.1001/jama.2020.17598 33201207

[9] Zlotta AR Ballas LK Niemierko A Lajkosz K Kuk C Miranda G . Radical cystectomy versus trimodality therapy for muscle-invasive bladder cancer: a multi-institutional propensity score matched and weighted analysis. Lancet Oncol. (2023) 24:669–81. doi: 10.1016/S1470-2045(23)00170-5 37187202

[10] Buksh O Almalki AM Khogeer A Al-Maghrabi J Alakraa M . A large inflammatory myofibroblastic tumor of the urinary bladder in a parturient treated by partial cystectomy: case report and literature review. Cureus. (2022) 14:1. doi: 10.7759/cureus.29556 PMC9595141 36312673

[11] Martinez-Trufero J Cruz Jurado J Gomez-Mateo MC Bernabeu D Floria LJ Lavernia J . Uncommon and peculiar soft tissue sarcomas: Multidisciplinary review and practical recommendations for diagnosis and treatment. Spanish group for Sarcoma research (GEIS - GROUP). Part I. Cancer Treat Rev. (2021) 99:102259. doi: 10.1016/j.ctrv.2021.102259 34311246

[12] Fachini Cipriani RF Cavalli AC Andrade JL Sfredo LR Martins da Silva IV de Souza Digner I . Inflammatory myofibroblastic bladder tumor: A very rare presentation. Urol Case Rep. (2021) 39:101863. doi: 10.1016/j.eucr.2021.101863 34631428 PMC8487991

[13] Khondakar NR Lee P McNeil BK . Gross hematuria in an adolescent secondary to a rare bladder tumor: A case report and review of inflammatory myofibroblastic tumors of the urinary bladder. Urology. (2022) 165:e39–45. doi: 10.1016/j.urology.2022.01.034 35123984

[14] Braham Y Migaou A Njima M Achour A Ben Saad A Cheikh Mhamed S . Inflammatory myofibroblastic tumor of the lung: A rare entity. Respir Med Case Rep. (2020) 31:101287. doi: 10.1016/j.rmcr.2020.101287 33251105 PMC7683262

[15] Nakano K . Inflammatory myofibroblastic tumors: recent progress and future of targeted therapy. Jpn J Clin Oncol. (2023) 53:885–92. doi: 10.1093/jjco/hyad074 37394916

[16] Lopez-Beltran A Cookson MS Guercio BJ Cheng L . Advances in diagnosis and treatment of bladder cancer. Bmj. (2024) 384:12. doi: 10.1136/bmj-2023-076743 38346808

[17] Kim KS Kim SH Cho HJ Sur HJ Choi YS . Holmium laser-assisted laparoscopic partial cystectomy for bladder cancer: a single-institutional pilot study with technical feasibility and short-term oncological outcome. BMC Cancer. (2022) 22:2. doi: 10.1186/s12885-022-09308-7 35189855 PMC8862280

[18] Fu J Fu F Wang Y . 1470 nm/980 nm dual-wavelength laser is safe and efficient for the en-bloc resection of non-muscle invasive bladder cancer: A propensity score-matched analysis. J Int Med Res. (2021) 49:3000605211065388. doi: 10.1177/03000605211065388 34939431 PMC8721717

[19] Lopez-Nunez O John I Panasiti RN Ranganathan S Santoro L Grélaud D . Infantile inflammatory myofibroblastic tumors: clinicopathological and molecular characterization of 12 cases. Modern Pathol. (2020) 33:576–90. doi: 10.1038/s41379-019-0406-6 31690781

[20] Wachter F Janeway KA . Comment on: Clinical, pathologic, and molecular features of inflammatory myofibroblastic tumors in children and adolescents: ROS1-fusion inflammatory myofibroblastic tumor: ROS1-fusion inflammatory myofibroblastic tumor. Pediatr Blood Cancer. (2023) 70:e29907. doi: 10.1002/pbc.29907 35920604

[21] Da M Qian B Mo X Xu C Wu H Jiang B . Inflammatory myofibroblastic tumors in children: A clinical retrospective study on 19 cases. Front Pediatr. (2021) 9:543078. doi: 10.3389/fped.2021.543078 34307241 PMC8295553

[22] Yamamoto H Yoshida A Taguchi K Kohashi K Hatanaka Y Yamashita A . ALK, ROS1 and NTRK3 gene rearrangements in inflammatory myofibroblastic tumours. Histopathology. (2016) 69:72–83. doi: 10.1111/his.2016.69.issue-1 26647767

[23] Rao N Iwenofu H Tang B Woyach J Liebner DA . Inflammatory myofibroblastic tumor driven by novel NUMA1-ALK fusion responds to ALK inhibition. J Natl Compr Canc Netw. (2018) 16:115–21. doi: 10.6004/jnccn.2017.7031 29439172

[24] Siemion K Reszec-Gielazyn J Kisluk J Roszkowiak L Zak J Korzynska A . What do we know about inflammatory myofibroblastic tumors? - A systematic review. Adv Med Sci. (2022) 67:129–38. doi: 10.1016/j.advms.2022.02.002 35219201

[25] Bertz S Otto W Denzinger S Wieland WF Burger M Stöhr R . Combination of CK20 and ki-67 immunostaining analysis predicts recurrence, progression, and cancer-specific survival in pT1 urothelial bladder cancer. Eur Urol. (2014) 65:218–26. doi: 10.1016/j.eururo.2012.05.033 22633802

